# Qiliqiangxin Affects L Type Ca^2+^ Current in the Normal and Hypertrophied Rat Heart

**DOI:** 10.1155/2012/131830

**Published:** 2012-03-26

**Authors:** Yidong Wei, Xiaoyu Liu, Lei Hou, Wenliang Che, Erlinda The, Muktanand Vikash Jhummon

**Affiliations:** ^1^Department of Cardiology, Shanghai Tenth People's Hospital of Tongji University, 301 Yanchang Road, Shanghai 200072, China; ^2^Department of Traditional Chinese Medicine, Shanghai Tenth People's Hospital of Tongji University, 301 Yanchang Road, Shanghai 200072, China

## Abstract

Qiliqiangxin capsule is newly developed Chinese patent drug and proved to be effective and safe for the treatment of patients with chronic heart failure. We compared the effects of different dose Qiliqiangxin on L type Ca^2+^ current (*I*_Ca-L_) between normal and hypertrophied myocytes. A total of 40 healthy Sprague—Dawley rats were used in the study. The rats were randomly divided into two groups (control group and hypertrophy group). Cardiac hypertrophy was induced by pressure overload produced by partial ligation of the abdominal aorta. The control group was the sham-operated group. After 1 month, cardiac ventricular myocytes were isolated from the hearts of rats. Ventricular myocytes were exposed to 10 and 50 *μ*mol/L Qiliqiangxin, and whole cell patch-clamp technique was used to study the effects of Qiliqiangxin on *I*_Ca-L_. The current densities of *I*_Ca-L_ were similar in control group (−12.70 ± 0.53 pA/pF, n = 12) and in hypertrophy group (−12.39 ± 0.62 pA/pF, n = 10). They were not statistically significant. 10 and 50 *μ*mol/L Qiliqiangxin can decrease *I*_Ca-L_ peak current 48.6%±16.8% and 59.0%±4.4% in control group. However, the peak current was only reduced 16.73%±8.03% by 50 *μ*mol/L Qiliqiangxin in hypertrophied myocytes. The inhibited action of Qiliqiangxin on *I*_Ca-L_ of hypertrophy group was lower than in control group. Qiliqiangxin affected L-type Ca^2+^ channel and blocked *I*_Ca-L_, as well as affected cardiac function finally. Qiliqiangxin has diphasic action that is either class IV antiarrhythmic agent or the agent of effect cardiac function.

## 1. Introduction

The traditional Chinese medicines have proven the safety and efficiency of herbs in the management of some diseases since ancient times. Qiliqiangxin capsule is newly developed Chinese patent drug and proved to be effective and safe by phase 3 clinic trial for the treatment of patients with chronic heart failure. It includes over 11 ingredients such as Ginseng, Radix Astragali, Aconite Root, Salvia Miltiorrhiza, and Semen Lepidii Apetali [1, 2]. Previous studies showed Qiliqiangxin capsule can improve heart function and decrease serum level of TNF-*α* and relieve inflammatory cell infiltration of myocardium in rats with adriamycin induced cardiomyopathy [3]. Nevertheless, the excitation-contraction coupling in the cardiac myocyte is triggered by the influx of Ca^2+^ through L type Ca^2+^ channel and inducing Ca^2+^ release from the sarcoplasmic reticulum [4, 5]. Blocking Ca^2+^ channel and reducing Ca^2+^ overload will be of benefit in the progress of heart failure. Qiliqiangxin should affect L type Ca^2+^ channel and further alter cellular Ca^2+^ regulation as well as effect the heart function finally. We compared the effects of different dose Qiliqiangxin on L type Ca^2+^ current (*I*_Ca-L_) between normal and hypertrophied myocytes and to comprehend the rational usage of Qiliqiangxin on hypertrophied myocytes in this study.

## 2. Material and Methods

### 2.1. Vegetal Material

Qiliqiangxin consists of Ginseng, Radix Astragali, Aconite Root, Salvia Miltiorrhiza, Semen Lepidii Apetali, Cortex Periplocae Sepii Radicis, Rhizoma Alismatis, Carthamus Tinctorius, Polygonatum Odorati, Seasoned Orange Peel, and Rumulus Ginnamomi [3] (Yiling Pharmaceutical Corporation, Shijiazhuang, China). The drug powder was dissolved with sterile water at the concentration of 2.67 g/mL. 10 *μ*moL/l and 50 *μ*mol/L Qiliqiangxin were prepared for the study. 

### 2.2. Study Models

A total of 40 healthy Sprague-Dawley rats (9–11-week old, either sex, weight 210 to 300 g) were used in the study. All the rats used in the following experiments were subject to the Guiding Principles for the Care and Use of Laboratory Animals and the Recommendations from the Declaration of Tongji University. The rats were randomly divided into two groups (control group and hypertrophy group). Cardiac hypertrophy was induced by pressure overload produced by partial ligation of the abdominal aorta by using the method described by Anderson [6–8]. The control group was the sham-operated group; the aorta was dissected without application of the ligation. After operation, both groups were fed up with normal fodder and tap water in different cages for one month.

### 2.3. Cardiac Ventricular Myocytes Isolation

Cardiac ventricular myocytes were isolated from the hearts of rats using previous protocols [9]. Briefly, hearts were rapidly excised and cycloperfused with low calcium Tyrode's solution containing 0.08% Collagenase, 0.006% Protease, and then get single ventricular myocyte. The single ventricular myocyte selected for study is rod shaped, had clear striations and smooth and glossy surface.

### 2.4. Whole Cell Patch Clamp

We recorded Ca^2+^ current in a Na^+^-free bath solution. To block outward K^+^ currents the bath contained (mM): 120 CsCL, 2 CaCl_2_,10 TEA, 5 4-AP, 1 MgCl_2_, 5 HEPES, 5 Glucose. PH = 7.4 (CsOH). The patch pipettes (borosilicate glass, 1.5–3 M*Ω*) were filled with the pipette solution (mM): 120 CsCl, 1 CaCl_2_, 10 HEPES, 5 Mg-ATP, 10 EGTA. PH = 7.2 (CsOH). All recordings are at room temperature. The external solution was filled with 95% O_2_ and 5% CO_2_. Ca^2+^ currents were elicited by voltage steps from −90 to +55 mV. Compensated series resistance was1.59 ± 0.20 M*Ω*. Cell capacitance averaged 26.9 ± 4.1 pF (*n* = 10 per group). To normalize for differences in total membrane area, current densities (in pA/pF) were calculated by dividing the total current by the membrane capacitance of the cell. Data were sampled at 10 kHz and filtered at 2 kHz by using an Axopatch 200A amplifier (Axon Instruments).

### 2.5. Statistical Analysis

pCLAMP 9.0 software was used for data acquisition and analysis values are presented as means ± S.D. Statistical comparisons between the different amiodarone concentrations groups were obtained by ANOVA. Comparisons between control and hypertrophied myocytes group means were performed with Student's *t*-test. Differences with *P* < 0.05 were considered significant, completed by SPSS 11.5 Statistically package. Concentration-response relationships were fit to the Hill equation to determine the concentration of drug required for 50% inhibition (IC_50_).

## 3. Results

### 3.1. Rats Characteristics

The rat hearts were significantly larger in hypertrophy group (810 ± 15 mg, *n* = 22) than in control group (730 ± 26 mg, *n* = 18). However, there was no difference in body weight between the two groups. Heart weight index (heart weight/body weight, HW/BW) and left ventricular weight index (left ventricular weight/body weight, LVW/BW) in hypertrophy group were greater than those in control group. They were statistically significant (Table 1).

### 3.2. Effects of Qiliqiangxin on *I*_Ca-L_

**Figure 1:**
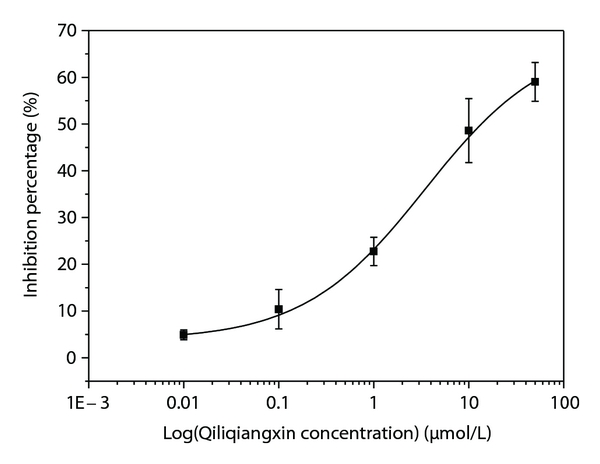
The concentration-dependent manner of Qiliqiangxin on *I*_Ca-L_ in cardiac myocytes of rat (IC_50_ = 10.38 *μ*mol/L).

**Figure 2:**
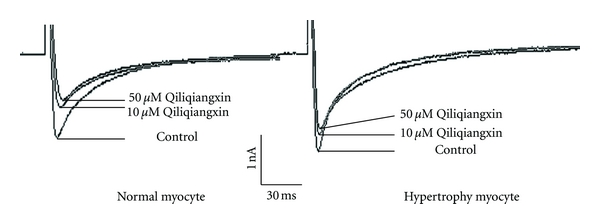
The effects of 10 and 50 *μ*mol/L Qiliqiangxin on *I*_Ca-L_ in normal and hypertrophied myocytes.

The current densities of   *I*_Ca-L_ were similar in control group (−12.70 ± 0.53 pA/pF, *n* = 12) and in hypertrophy group (−12.39 ± 0.62 pA/pF, *n* = 10). They were not statistical significant. Qiliqiangxin obviously decrease *I*_Ca-L_ of normal myocytes and represented a concentration-dependent manner. Its IC_50_ was 10.38 *μ*mol/L (Figure 1). 10 and 50 *μ*mol/L Qiliqiangxin can decreased *I*_Ca-L_ peak current 48.6% ± 16.8% and 59.0% ± 4.4% in control group. Interestingly, *I*_Ca-L_ represented insensitivity for Qiliqiangxin in hypertrophied myocytes. The peak current was only reduced 16.73% ± 8.03% by 50 umol/L Qiliqiangxin. Therefore, the inhibited action of Qiliqiangxin on *I*_Ca-L_ of hypertrophy group was lower than in control group (Figure 2).

## 4. Discussion

Cardiac hypertrophy is associated with a significantly increased risk of cardiovascular morbidity and mortality that were frequently induced by electrical remodeling and arrhythmogenesis. The antiarrhythmic research was most based on normal myocytes, and whether they have same action on pathosis myocytes was unknown. As a result, means of treating hypertrophy-associated arrhythmias remain disappointingly ineffective. So far, there were four main classes of antiarrhythmic agents. Class IV agents are slow calcium channel blockers and decrease conduction through the AV node. They shorten the plateau of the action potential and reduce the contractility of the heart. Class IV agents may be inappropriate in cardiac hypertrophy treatment. Nevertheless, blocking Ca^2+^ channels and reducing Ca^2+^ overload will be of benefit in the progress of cardiac hypertrophy.

In pressure overload hypertrophy models, we found that the currents amplitude of *I*_Ca-L_ on hypertrophied myocytes were higher than those in control. But the current densities were similar because of the swelling volume of hypertrophied myocytes. Acute application of Qiliqiangxin does inhibit *I*_Ca-L_ in normal cardiac myocytes. IC_50_ was 10.38 *μ*mol/L. 10 and 50 *μ*mol/L Qiliqiangxin can, respectively, decreased 48.6% ± 16.8% and 59.0% ± 4.4% of the peak current of *I*_Ca-L_ in control group. To compare with the hypertrophy group, *I*_Ca-L_ showed different effects of Qiliqiangxin. Interestingly, the peak current was only reduced 16.73% ± 8.03% by 50 *μ*mol/L Qiliqiangxin. The inhibited action of Qiliqiangxin on *I*_Ca-L_ of hypertrophy group was lower than in control group. In other words, *I*_Ca-L_ represented more insensitivity for Qiliqiangxin in hypertrophied cardiac myocytes. Qiliqiangxin displayed the insensitiveness that may be facilitated for its utilization in cardiac hypertrophy and heart failure. Because it partly blocked *I*_Ca-L_ and did not weaken myocardial contractility basically. 10 *μ*mol/L Qiliqiangxin obviously decreased 48.6%±16.8% of the peak current of *I*_Ca-L_ in normal cardiac myocytes, which made it reserve antiarrhythmic activity as class IV agents. That also signifies we should deal with difference between hypertrophied heart and normal heart when we use Qiliqiangxin in the clinic.

Qiliqiangxin includes over 11 ingredients. The mechanism of the antiarrhythmic action is complex and not completely understood. It is hard to prove which herb has mainly contributed to the effect on L type Ca^2+^ channel. Recently, ShenSongYangXin capsule, a traditional Chinese herb, has been reported to effectively block *I*_Ca-L_ [10]. Zhao et al. had reported that Radix Astragali effectively protected against cardiac dysfunctional and morphological aberrations in experimental myocardial infarction [11]. Aconite Root was proved to have positive inotropic, positive chronotropic, vasodilation, and diuretic effects in the management of congestive heart failure [12]. Qiliqiangxin is composed of Radix Astragal, Aconite Root and parts of Shensong Yangxin which are the main active constituents of Qiliqiangxin. In the cardiac function, the excitation-contraction coupling of cardiac myocyte is triggered by Ca^2+^ influx through L-type Ca channels [4, 5]. Ca^2+^ influx activates calmodulin kinase that may activate transcription factors and cAMP response element binding protein (CREB). CREB promoted several cytokine secretions such as interleukin-10 (IL-10) [13, 14]. Inflammatory cytokines mainly derived from cardiac myocytes were involved in the progression of heart failure [15]. A proinflammatory cytokine (TNF-*α*) has been linked to accelerate myocardial necrosis and deteriorated cardiac function. Serum level of TNF-*α* in patients with chronic heart failure increased and correlated with poor cardiac performance [16]. IL-10 and TNF-*α* induced left ventricular remodeling and dysfunction in the failing heart [17]. Qiliqiangxin may improve cardiac function of rats with MI through regulation the balance between TNF-*α* and IL-10 [3]. Blocking Ca^2+^ channels and reducing Ca^2+^ influx as well as weakening myocardial contractility will be the key point in the progress of cardiac hypertrophy and heart failure. The mechanism underlying the beneficial effects of Qiliqiangxin may involve the regulation of Ca^2+^ channel and reduce Ca^2+^ influx. Meantime, it influences several cytokine secretions indirectly. We concluded Qiliqiangxin affected L-type Ca^2+^ channel and blocked *I*_Ca-L_, as well as affected cardiac function finally. Qiliqiangxin has diphasic action that is either class IV antiarrhythmic agent or the agent of effect cardiac function.

### 4.1. Study Limitations

This study was focused on the effect of Qiliqiangxin in *I*_Ca-L_. Further studies should be on the regulation of Na^+^ and K^+^ channels.

## Figures and Tables

**Table 1:** The measurement of rats basic characteristics.

	BW (g)	HW (mg)	LVW (mg)	HW/BW (mg/g)	LVW/BW (mg/g)
Control	237 ± 23	730 ± 26	507 ± 48	2.67 ± 0.10	2.01 ± 0.15
Hypertrophy	229 ± 18	810 ± 15*	672 ± 50*	3.43 ± 0.15*	2.63 ± 0.19*

*Notation*. *P < 0.05, compared to control group. BW: body weight, HW: heart weight, left ventricular weight: LVW.
